# Adult beliefs about cognitive development vary across experience and expertise: A focus group study

**DOI:** 10.1371/journal.pone.0272254

**Published:** 2022-08-01

**Authors:** Samantha P. Hutchinson, Erica H. Wojcik

**Affiliations:** Department of Psychology, Skidmore College, Saratoga Springs, New York, NY, United States of America; University of Stavanger: Universitetet i Stavanger, NORWAY

## Abstract

The purpose of this study was to explore North American adult beliefs and perspectives on how young children develop early cognitive, language, and word learning skills, and how these beliefs vary depending on experience and expertise. While there is a body of literature that uses questionnaires to assess beliefs about how children develop, traditional rating scales (e.g., Likert scales) may miss the nuances of how people think about child development. Thus, we ran six in-person focus groups, differing in parenthood status and expertise, to learn how various adults talk and reason about cognitive development. Questions throughout the focus group sessions were aimed at determining the quality and origins of participants’ beliefs. Four main patterns emerged: developmental psychologists who were also parents were the most certain in their statements, parents used more anecdotes than non-parents, non-parents were more likely to talk about development as controllable compared to parents, and participants in all groups frequently referred to environment-based influences on development. Together, the results suggest that many adults are uncertain about how children develop and that there are differences in how parents and non-parents reason about development. These findings have implications for how we interpret past survey results and motivate future studies about how experience with children changes adult beliefs and reasoning about child development.

## Introduction

Perspectives on how children learn, develop, and behave vary across and within populations and communities [1] and individual beliefs about child development affect how parents, caretakers, educators, and communities nurture their children [2]. For example, parents are more likely to initiate conversational turns with their infant if they think that language input influences language development [3]. Additionally, parents read to their children more often if their beliefs about emergent literacy align with the current scientific consensus [4].

Much of the research on beliefs about child development focuses on the relationship between parent beliefs and their child’s developmental or academic milestones. Mothers who believe that the environment can positively affect child developmental outcomes initiate high-quality linguistic turns with their children, which in turn correlates with more advanced syntactic skills [5]. Other studies have similarly found that parents’ beliefs about the role of the environment in their child’s development affects how they interact with their children ([3, 6–11], see [12] for a review). These studies substantiate the notion that child development is not an isolated activity; it happens in the context of interactions with caregivers, which in turn are influenced by caregivers’ own beliefs about child development.

Previous studies have demonstrated ways in which parental beliefs affect child outcomes across various domains, however, fewer studies investigate the quality and origins of these beliefs. Why do parents hold certain beliefs about early cognitive development? What experiences led to those beliefs? How confident are they in their beliefs? A better understanding of adult beliefs about child development will elucidate how both societal and individual perspectives on development affect child outcomes. It will also enable developmental scientists to better communicate their areas of expertise to the public and reveal how to best empower caregivers, policymakers, and voters with the knowledge they need to provide a rich early environment for their children.

### Measuring adult beliefs about development

The studies cited above focus on how adult beliefs about child development relate to developmental outcomes. This area of research overwhelmingly uses self-report measures and Likert scale surveys to assess beliefs about child development. However, Likert scale surveys have weaknesses that may lead researchers to misunderstand aspects of beliefs and cognitions. First, because researchers determine the survey questions, there may be topics or attitudes that are salient to many people but are not captured in the data. Second, by forcing participants to choose a categorical answer, researchers do not learn about the certainty participants have about their responses. Because researchers are most often interested in how beliefs affect behavior across time and contexts, we must understand if beliefs around child development are strong and stable, or if they are weak and malleable. Thus, the current study used interactive focus groups to more fully explore how adults talk and reason about child development.

One example of how focus groups can help us better comprehend adult beliefs about child development is in our understanding of how people think about the roles of genes and the environment. Parents who hold stronger beliefs about the genetic origins of development tend to have a lower perceived sense of control over their child’s behavior [13]. Beliefs about the role of genetics and the environment in development have often been studied by directly asking participants if they think certain traits are genetic/innate or environmental/learned [14, 15]. For example, one recent study asked adult participants a series of questions about early cognitive skills, such as face perception, to assess whether they believed the skills were innate or learned [15]. Significantly more people endorsed a learning, or empiricist, perspective across all measured abilities. This effect held both in a US sample and in a sample from India, as well as with school-aged children, suggesting that the effect was not influenced by enculturation.

### Using focus groups to expand our understanding of adult beliefs

However, we do not know how people typically talk to each other about the roles of genetics and learning in child development. For example, what educational sources, if any, do they draw on when discussing the roots of development? What types of personal experiences influence their beliefs? Are their beliefs strong, or do they not think about concepts like genetics and innateness often? Additionally, studies have found that many people embrace genetic essentialism, or the idea that one’s genes control their fate [16]. How do people apply ideas of controllability and genetic essentialism to child development? By asking open-ended questions and encouraging discussion in a focus group, we can better understand how people talk and think about child development, without imposing our own assumptions.

Additionally, most studies that investigate beliefs about child development have been conducted with parents. While there are theoretical reasons for this decision—often studies are interested in how beliefs translate to parent-child interactions—it leads to a lack of understanding about how people without children think about child development. Since all people have a role in determining the environment that children grow up in, either by taking care of relatives or friends, or by voting for policies related to education and childcare, we must also understand how non-parent populations think about child development.

### The current study

The current study presents a qualitative analysis of focus groups conducted across different populations to understand how adults think about cognitive development. The theoretically motivated sample for the current study was selected based on previous research on the nature of adult beliefs about child development [17]. Miller suggested that differing beliefs about cognitive development may stem from varying degrees of social interaction and experience with children. For example, non-parents may have different beliefs than parents and parents may assess cognitive abilities in children differently than cognitive scientists. Furthermore, an individual’s beliefs may change after becoming a parent as they have more direct and continuous experience with development. However, the literature is lacking in studies that compare the beliefs of these various groups of people. Therefore, the current study aims to describe and compare a set of four types of participants across two dimensions: expertise in development (developmental psychologists vs. non-specialists) and parenthood status (parents vs. non-parents).

Through focus groups with each type of participant, origins of beliefs and perspectives on three distinct categories were discussed: (1) how young children develop cognitive skills, such as reasoning and problem solving, (2) language acquisition, such as how children begin to understand what words mean and learn grammatical concepts, and (3) word learning in terms of how children learn their first words. Participants’ responses were coded to reveal emergent themes in the discussions, providing exploratory, qualitative data to help generate novel hypotheses about sources of variability in adult beliefs about child development.

## Materials and methods

### Participants

A total of six focus groups were conducted across two dimensions: expertise in development (developmental psychologists vs. non-specialists) and parenthood status (parents vs. non-parents). Specifically, we ran two separate groups of parents, two separate groups of students, one group of developmental psychologists who were parents, and one group of developmental psychologists who were not parents. This focus group study only recruited women. We restricted all parental groups to parents with children aged 0–10 years because of the study’s focus on early childhood development.

Parent participants were recruited from an upstate New York Community and compensated with $10, college students were compensated with course credit, and developmental psychologists (both parents and non-parents) were recruited at an academic conference and compensated $10. Only women participants were recruited for the results to be comparable to past research, which overwhelmingly reports on mother-child interactions and beliefs [3, 5, 7–9, 11, 18]. The final sample consisted of n = 32 adult women participants (18 years or older). Race and ethnicity varied across the sample: 72 percent white, 6 percent Hispanic or Latino, 2 percent Asian, and 16 percent identified as two or more races/ethnicities. All participants held some college degree or higher education (see Table 1 for more demographic information). The sample size was based on recommendations from David L. Morgan’s book, *Focus Groups as Qualitative Research* [19]. Written consent was obtained from all participants according to the Skidmore College Institutional Review Board (#1907–821).

**Table 1:** Demographics of study sample.

Question	Response options	Students (count /percent)	Parents (count /percent)	DP_Ps (count /percent)	DP_NPs (count /percent)	Total (count /percent)
**Your race/ethnicity**	n	15	9	3	5	32
	Asian	2 / 13	0 / 0	0 / 0	0 / 0	2 / 6
	American Indian or Alaska Native	0 / 0	0 / 0	0 / 0	0 / 0	0 / 0
	Black or African American	0 / 0	0 / 0	0 / 0	0 / 0	0 / 0
	Hispanic or Latino	1 / 7	0 / 0	0 / 0	1 / 20	2 / 6
	White	8 / 53	9 / 100	2 / 67	4 / 80	23 / 72
	Native Hawaiian or Other Pacific Islander	0 / 0	0 / 0	0 / 0	0 / 0	0 / 0
	2 or more races/ethnicities	4 / 27	0 / 0	1 / 33	0 / 0	5 / 16
**Highest education you have achieved**	n	15	9	3	5	32
	Some high school	0 / 0	0 / 0	0 / 0	0 / 0	0 / 0
	High school degree/GED	0 / 0	0 / 0	0 / 0	0 / 0	0 / 0
	Some college	15 / 100	0 / 0	0 / 0	0 / 0	15 / 47
	Associates degree/2 yr. Technical degree	0 / 0	1 / 11	0 / 0	0 / 0	1 / 3
	Bachelor’s degree	0 / 0	1 / 11	0 / 0	0 / 0	1 / 3
	Master’s degree	0 / 0	4 / 44	1 / 33	0 / 0	5 / 16
	PhD	0 / 0	3 / 33	2 / 67	5 / 100	10 / 31
**Child/children’s age (years)**	n	NA	16	3	NA	NA
	0–2 years	NA	3 / 19	3 / 100	NA	NA
	3–5 years	NA	5 / 31	0 / 0	NA	NA
	6–8 years	NA	4 / 25	0 / 0	NA	NA
	9–11 years	NA	2 / 13	0 / 0	NA	NA
	12+ years	NA	2 / 13	0 / 0	NA	NA
**Child/children’s gender**	n	NA	16	3	NA	NA
	Male	NA	11 / 69	0 / 0	NA	NA
	Female	NA	5 / 31	3 / 100	NA	NA
**Do your children speak a language other than English?**	n	NA	16	3	NA	NA
	Yes	NA	1 / 6	1 / 33	NA	NA
	No	NA	15 / 94	2 / 67	NA	NA
**Do you speak a language other than English?**	n	15	NA	NA	5	NA
	Yes	5 / 33	NA	NA	3 / 60	NA
	No	10 / 67	NA	NA	2 / 40	NA
**Did you take any psychology courses in high school/college?**	n	15	9	NA	NA	NA
	Yes	13 / 87	7 / 78	NA	NA	NA
	No	2 / 13	2 / 22	NA	NA	NA
**Did you take any developmental psychology or child development courses?**	n	15	9	NA	NA	NA
	Yes	5 / 33	1 / 11	NA	NA	NA
	No	10 / 67	8 / 89	NA	NA	NA

*Note*. DP_Ps is the Developmental Psychologist Parent group and DP_NPs is the Developmental Psychologist Non-Parent group. Cells with NA indicate that a particular question was not asked to that group due to the population characteristics.

### Procedure

Each of the six focus groups consisted of 2–13 participants, one moderator, and one note-taker based on the guidelines in David L. Morgan’s book, *Focus Groups as Qualitative Research* [19]. Upon arrival, each participant filled out a consent form and a demographics questionnaire. The consent form was the same for all participants and the demographics questionnaire varied slightly by participant type. For example, parents and developmental psychologists who were also parents were asked if their children speak a language other than English, whereas students and developmental psychologists who were not parents were only asked if they themselves spoke a language other than English. The one-hour in-person session was conducted by a student moderator who used a set list of questions to guide each discussion (see S1 File). The primary investigator (PI) also attended each focus group session and encouraged conversation among participants by elaborating on questions if needed. Both the moderator and the PI introduced themselves to the group at the beginning of each session. The questions followed a general format of three main topics: cognitive development, language development, and word learning skills. Questions were aimed at identifying possible origins of adult beliefs about these topics. While all the questions were discussed in each session, they did not always follow the same order, allowing for a more naturally flowing group discussion. Participants were encouraged to freely contribute their thoughts to the discussion, in no specified participant order, under the direction that there were no correct answers to any of the prompted questions. After each session, they were debriefed on the purpose of the study. All sessions were recorded via a video camera, with a backup audio recorder as well. While first names were used during each session, transcripts and coded data did not include any names or identifiable information. All recordings were locked in the lab at all times, only to be viewed by trained coders. All study procedures and protocols were approved by the Institutional Review Board.

### Data analysis

All focus group sessions were transcribed and coded by trained researchers using ELAN 5.0.0 Transcription Software [20]. Transcriptions were broken up into utterances, which were defined as a complete idea from one participant. Each utterance was then coded according to the Coding Key, which was developed in a meeting with the transcribers using an inductive method where general topics from the transcriptions (Code Category) were broken down into more specific components (Code Level; see Table 2). The Coding Key reflected major themes that were represented across the groups. A total of 60 utterances (8.9 percent) were marked NA because they were not relevant across any coding categories and thus not included in the analyses. An example of an utterance marked NA is “Your daughter and my daughter should hang out.” A second coder coded a random sample of 20 percent of the participant utterances to check inter-rater reliability. There was 86% agreement between coders and the average Cohen’s Kappa was 0.80. The Cohen’s Kappa for all four categories was also high (Certainty: 0.86, Statement Type: 0.90, Control: 0.72, and Etiology: 0.71).

**Table 2:** Coding key.

Code Category	Code Level	Code Name	Description
Certainty	Certain	Certain	Any statement that is not quantified; a direct, confident statement
	Uncertain	Uncertain	Any statement that expresses doubt or uncertainty about its veracity, e.g., uses phrases like "I don’t know" or "maybe"
Statement Type	Anecdote	Anecdote	Story or statement about one or multiple children (either directly observed or heard about)
	General	General	Generic statement about children/development, e.g., "Children learn by X"
	Comparison	Comparison	Comparing specific child/incident to overall population/children in general
Control	Controllable	Controllable	Something that is changeable/one can affect in any way by parents (can the parent directly control the situation?)
	Uncontrollable	Uncontrollable	Something that is not changeable/under one’s control (parent cannot control the situation)
Etiology	Genetic	Genetic	Determined by genetic makeup, in the child’s DNA, innate from birth (nature)
	Environmental	Environmental	Influenced by factors around them, e.g., parenting, SAS, location (nurture)
	Interaction	Interaction	Both Genetic and Environmental
Mechanism	Trial and Error	TrialError	Children learn by trying things out, making mistakes and correcting them
	Observation	Observation	Children learn by watching or listening passively
	Direct Instruction	Instruction	Children learn by being explicitly taught by another person
	Repetition	Repetition	Children learn by doing something over and over
	Mimicking	Mimicking	Children learn by copying what they see/hear
	Reinforcement	Reinforcement	Children learn by getting positive feedback from another person
	Other	Other	Any other statement about learning mechanism that is not one of the above
Topic	Cognitive Development	Cognitive	Refers to cognition (problem solving, etc., NOT language)
	Language	Language	Refers to language skills or development
	Words	Words	Refers to word learning or use
	Temperament Personality	Temperament	Refers to a child’s personality or general temperament, including preferences/proclivities
	Social	Social	Refers to child’s social interactions
	Motor	Motor	Refers to motor skills or development, such as walking, crawling etc.
	Other	Other	Any statement about another domain of development

## Results

Using the Coding Key, participant responses were coded in accordance with the six major themes (Code Categories) that emerged from the focus groups: Certainty, Statement Type, Control, Etiology, Mechanism, and Topic. Each participant utterance was given a Code Level for each Code Category (see Table 2). For example, under the Code Category of Certainty, the Code Levels included Certain and Uncertain. The category “Topic” was used as an organizational tool, and a similar pattern of results was observed across all topics, so this Code Category is not discussed further.

### Theme 1: Certainty

**Fig 1:**
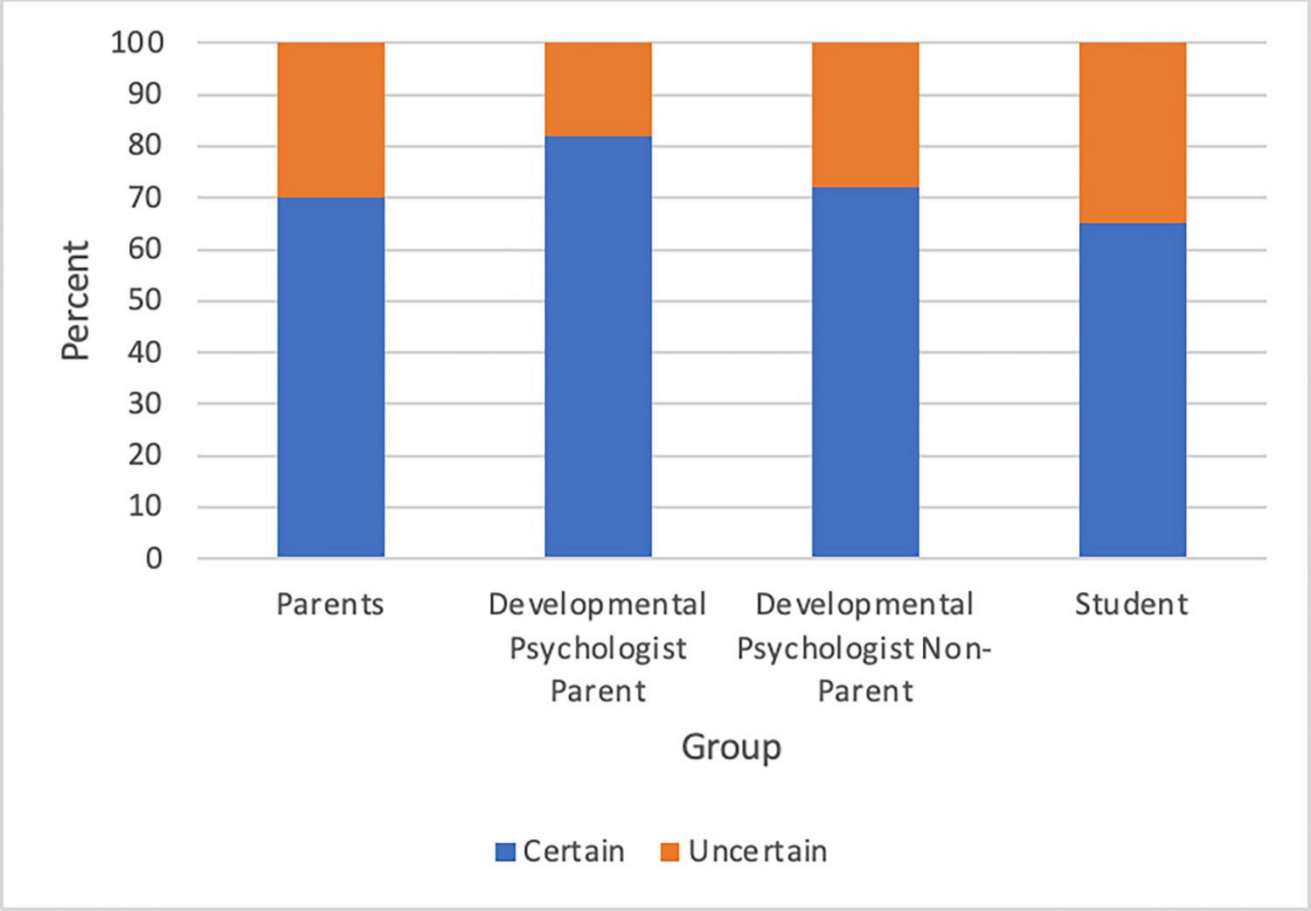
Participant certainty by group.

Utterances were coded as Certain if they were direct, confident statements and were coded as Uncertain if they expressed doubt. The Developmental Psychologist Parent group was the most certain in their statements, with 82% of their utterances coded as Certain (see Fig 1 and Table 3; all reported averages are on the utterance level, such that each utterance is weighted equally). On the topic of word learning skills, one developmental psychologist who was a parent expressed certainty when she described her experience with her child’s first word:

“My kid’s first word was doggie, and I 100 percent attribute that to our dog. It’s possibly the most salient aspect of our household.”

**Table 3:** Frequency of participant utterances for all levels.

Category	Group	Level	Frequency (count)	Frequency (%)	Selected Examples
**Certainty**	Parents	Certain	133	70	“I think behaviorally there’s definitely fits and starts and regressions and in terms of appropriate behavior and stuff like that.”
		Uncertain	58	30	
	DP_Ps	Certain	150	82	
		Uncertain	32	18	“I think the reason I am so hesitant to come to genetics is so personally it feels very hard to explain this higher order thing from a genetic mechanistic explanation. So, I’m just very reticent to make that connection.”
	DP_NPs	Certain	52	72	
		Uncertain	20	28	
	Student	Certain	104	65	
		Uncertain	57	35	
**Statement Type**	Parents	Anecdote	121	65	“My 6-year-old now picks up stuff that her brother and sister do as teenagers and she does teenager stuff sometimes. So, it’s good and bad, but she also does multiplication, so I mean it’s both so ya know.”
		General	56	30	
		Comparison	10	5	“I mean, I feel like it’s progressing at what would be considered a healthy—probably developmentally appropriate. Like, he’s doing developmentally appropriate things for his age.”
	DP_Ps	Anecdote	104	57	
		General	71	39	
		Comparison	6	3	
	DP_NPs	Anecdote	4	6	
		General	60	86	
		Comparison	6	9	
	Student	Anecdote	30	19	
		General	104	65	“But also, there are kids that skip steps, that are like prodigies or are like excelling in areas and so they’ll skip steps that some people would do.”
		Comparison	26	16	
**Control**	Parents	Controllable	65	49	
		Uncontrollable	67	51	
	DP_Ps	Controllable	49	49	
		Uncontrollable	50	51	“It’s about the predictability too like thinking that everything is from the environment means that you should be able to shape your kids in a certain way. And I now absolutely don’t think that I have that much control to shape her. Like she’s gonna be her own person.”
	DP_NPs	Controllable	17	63	
		Uncontrollable	10	37	
	Student	Controllable	72	80	“And also, like sometimes people correct kid’s language, like if they use like the wrong grammar. Like no that’s not what you’re supposed to say!”
		Uncontrollable	18	20	
**Etiology**	Parents	Genetic	36	27	“So, the cultural circumstances are very similar, but the kids are very different. So yeah, it’s probably all genetics.”
		Environmental	77	58	
		Interaction	20	15	
	DP_Ps	Genetic	42	40	
		Environmental	52	50	
		Interaction	10	10	
	DP_NPs	Genetic	8	15	
		Environmental	34	65	“I think that there are certain things, so I study caregiver-infant interactions, caregivers modify their behaviors in certain ways that help facilitate learning and attention to what they’re doing.”
		Interaction	10	19	
	Student	Genetic	14	10	
		Environmental	107	73	
		Interaction	25	17	“Like my brother and I like grew up in obviously like the same environment, but like he’s very like naturally good at like retaining information, where like I am like not as naturally good at that. Um, but I don’t know cuz our genes are probably similar as well, so it’s just like I don’t know.”
**Mechanism**	Parents	Trial and Error	3	3	
		Observation	2	2	
		Direct Instruction	5	4	
		Repetition	1	1	“I think repetition. So, trial and error and the repeating, but also repeating it correctly a few times, once it finally worked out.”
		Mimicking	8	7	“I think it’s pretty much pure mimicry to be honest. How kids pick these things up. I just think they repeat what they hear.”
		Reinforcement	4	3	
		Other	92	80	
	DP_Ps	Trial and Error	1	9	“Yeah, it’s a lot of like testing things out and seeing what happens and seeing what reaction they get from their environment or from other people around them. Yeah, like trial and error.”
		Observation	0	0	
		Direct Instruction	1	9	
		Repetition	0	0	
		Mimicking	0	0	
		Reinforcement	5	45	“Yeah I think she followed that, but I also think we, I also wonder if we reinforced just things like the shift from babbling to word learning in a way, like if she goes ’mamasa’, oh are you saying mama? And that’s like giving her cues from the environment to reinforce that coupling.”
		Other	4	36	
	DP_NPs	Trial and Error	2	6	
		Observation	9	26	
		Direct Instruction	3	9	
		Repetition	0	0	
		Mimicking	0	0	
		Reinforcement	1	3	
		Other	20	57	
	Student	Trial and Error	3	3	
		Observation	20	19	“I think that kids might watch the way that your mouth moves, or when you speak, um say that like you’re saying something and you’re picking an item up, they make those associations rather at like a young age, and they remember them. So, with like baby steps, they just put things together.”
		Direct Instruction	21	20	“I think also um it comes from parents that explain like why things are like, like if there’s a punishment like why it is like um authoritative parents.”
		Repetition	11	11	
		Mimicking	7	7	
		Reinforcement	14	14	
		Other	27	26	“Yeah, I think privilege plays a big role too because some families are, don’t have the same access to the same kinds of schools that kids who are developing quicker are able to.”

*Note*. DP_Ps is the Developmental Psychologist Parent group and DP_NPs is the Developmental Psychologist Non-Parent group. And one example of each Level was provided across groups.

Students were the most uncertain in their statements with a total of 35 percent Uncertain utterances, leaving only 65 percent of their statements coded as Certain (see Table 3). When on the topic of language development in relation to environmental influences, one student expressed uncertainty:

“My brother and I grew up in obviously the same environment, but like he’s very naturally good at retaining information, where I am not as naturally good at that. Um, but I don’t know our genes are probably similar as well, so it’s just like I don’t know.”

### Theme 2: Statement type

**Fig 2:**
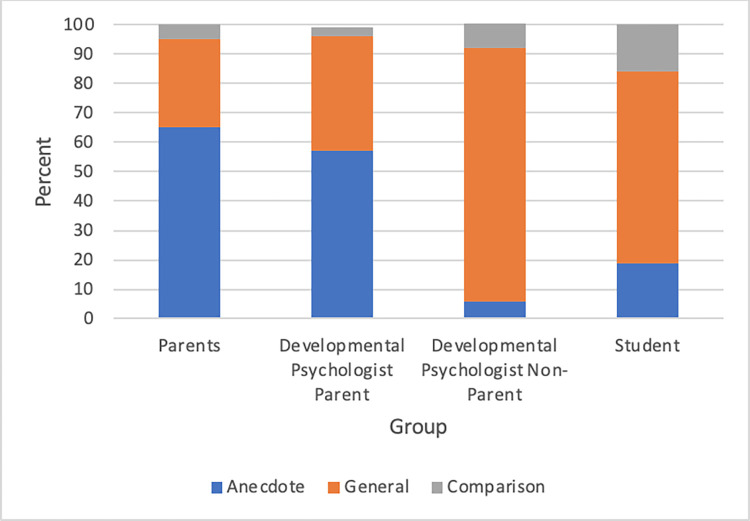
Participant statement types by group.

This category serves to represent the types of responses that participants gave. General Statements were defined as any generic statement about children and child development. Anecdotes were defined as statements, either directly observed, heard about, or experienced, involving at least one specific child. Any statement comparing a specific child to the general population of children was coded as a Comparison. The Developmental Psychologist Non-Parent group used the most General Statements, the Parent group used the most Anecdotes, and the Student group made the most Comparisons (see Fig 2). Overall, parents (both the Parent and Developmental Psychologist Parent groups) had higher percentages of Anecdotes than non-parents (Students and Developmental Psychologist Non-Parent).

Utterances by participants in the Developmental Psychologist Non-Parent group consisted of 86 percent General Statements (see Fig 2 and Table 3). One developmental psychologist who was not a parent expressed a General Statement on the topic of the role of genetics in child development:

“Yeah, and I guess if you take things, like sort of going that way, children that have some kind of developmental delay or if you’re not able to hear or things like that, that’s obviously going to be, that can be genetically determined and is going to affect the way that you interact with the world. So, kind of basic perceptual processes are going to be relevant and probably somewhat genetically tied.”

Participants who were parents used the most anecdotes: Parent group utterances were 65 percent Anecdotes and Developmental Psychologist Parent group utterances were 57 percent anecdotes (see Fig 2 and Table 3). One parent commented on her experience with her child’s language learning:

“Because our daughter lives with two English professors and we read to her all the time and we have conversations using complicated language around her all the time and then I was also a really early talker and I recognize some of myself in her. And my mom said she sees a lot of me in her when I was younger. But then my dad was an editor, and my mom was a language teacher, so I don’t know how to disentangle those. Yeah, so how much of her language learning has to do with that—I don’t know how to separate those things in our case.”

Students made the most comparisons (16 percent, see Fig 2 and Table 3). One student talked about how the process of repetition can reinforce language and word learning in children by providing an example from her childhood:

“I think that it starts out like a parent when they see a dog—like I know my mom always used to point out and say like oh it’s a dog—and just the repetition of hearing that lots of times would probably create those mental schemas.”

### Theme 3: Control

**Fig 3:**
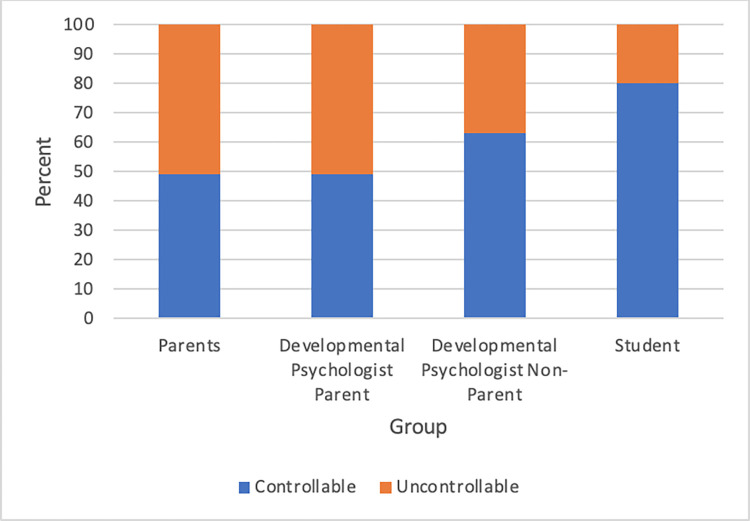
Participants’ perceived controllability by group.

The Control category creates a distinction between statements that conveyed that a child’s development was Controllable versus Uncontrollable. Controllable statements were defined as statements in which a child’s behavior is changeable or that a person can directly manipulate their development. Uncontrollable statements were defined as those that indicated that no person in the environment could control that aspect of development. The Student groups used the most statements that indicated Controllable outcomes for child development and the Parent groups (including Parents and Developmental Psychologist Parents), had more of an even split between Controllable and Uncontrollable statements (see Fig 3).

The Student groups made Controllable utterances 80 percent of the time (see Table 3). One student suggested that children learn from their environment, specifically from direct instruction and observation from one’s parents:

“I think that’s more environment. I guess more of like your parents teaching you and kind of just reading to you and like showing you what’s what. I think that’s more the environment, not genetics.”

In both the Parent group and the Developmental Psychologist Parent group, Controllable statements made up 49 percent and Uncontrollable statements were 51 percent of the utterances (see Table 3). One parent expressed an uncontrollable statement about the path of child development:

“I had something similar, that like you know even kids within the same family may be on a different path of development and that it’s really up to us to like to guide them, but we can’t control almost like any of the factors involved in that.”

In the Developmental Psychologist Parent group, a Controllable statement was expressed by a mother who described the process of teaching her child a new word:

“So, I can tell you how I taught my child ’more’. It was with blueberries, and it was actually to sign ’more’ first. My child loves berries, so I would give her a blueberry and I would say do you want more? *signs more* and then I would give her a blueberry and just over time she learned ’more’, and it then led to other words.”

### Theme 4: Etiology

**Fig 4:**
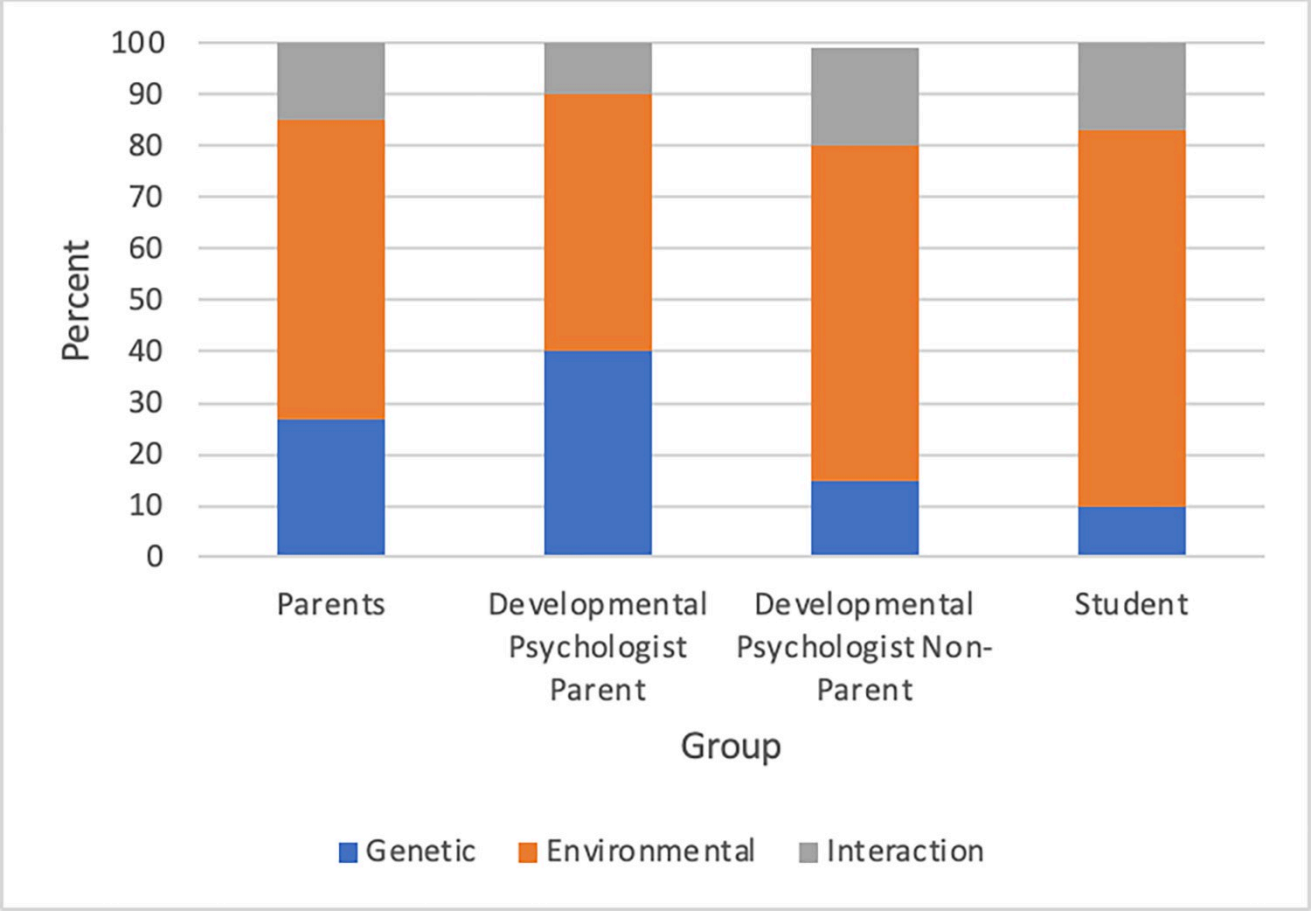
Participant responses on the etiology of development by group.

The Etiology category captured whether statements indicated that the environment, genetics, or an interaction of the two was the cause of a child’s development. Environmental statements were defined as those that suggested that behavior was influenced by external factors, while Genetic statements were those that indicated that behavior was influenced by one’s genetic makeup, or that it was internal and innate. Statements coded as Interaction included both environmental and genetic factors. Participants who were not parents (Student and Developmental Psychologist Non-Parent groups) demonstrated more Environmental statements; however Environmental statements were higher in general across groups (see Fig 4).

Student statements were 73 percent Environmental and Developmental Psychologist Non-Parent statements were 65 percent Environmental (see Table 3). A participant in the Developmental Psychologist Non-Parents group described how children learn from their environment:

“I think that there are certain things, so I study caregiver-infant interactions, caregivers modify their behaviors in certain ways that help facilitate learning and attention to what they’re doing.”

One participant in the Parent group discussed how her own children displayed similarities to her husband’s behavior as a child, indicating a Genetic etiology:

“Ok so um, I’ll say that like both of my boys seem to have, especially my 8-year-old with um he’s got ADHD and anxiety and whatever else we’re still figuring it out. But like his experiences and the way that he seems to think about things, it very much matches my husband’s experience as a child. So, I feel like there’s some component that has to do with genetics.”

### Theme 5: Mechanism

**Fig 5:**
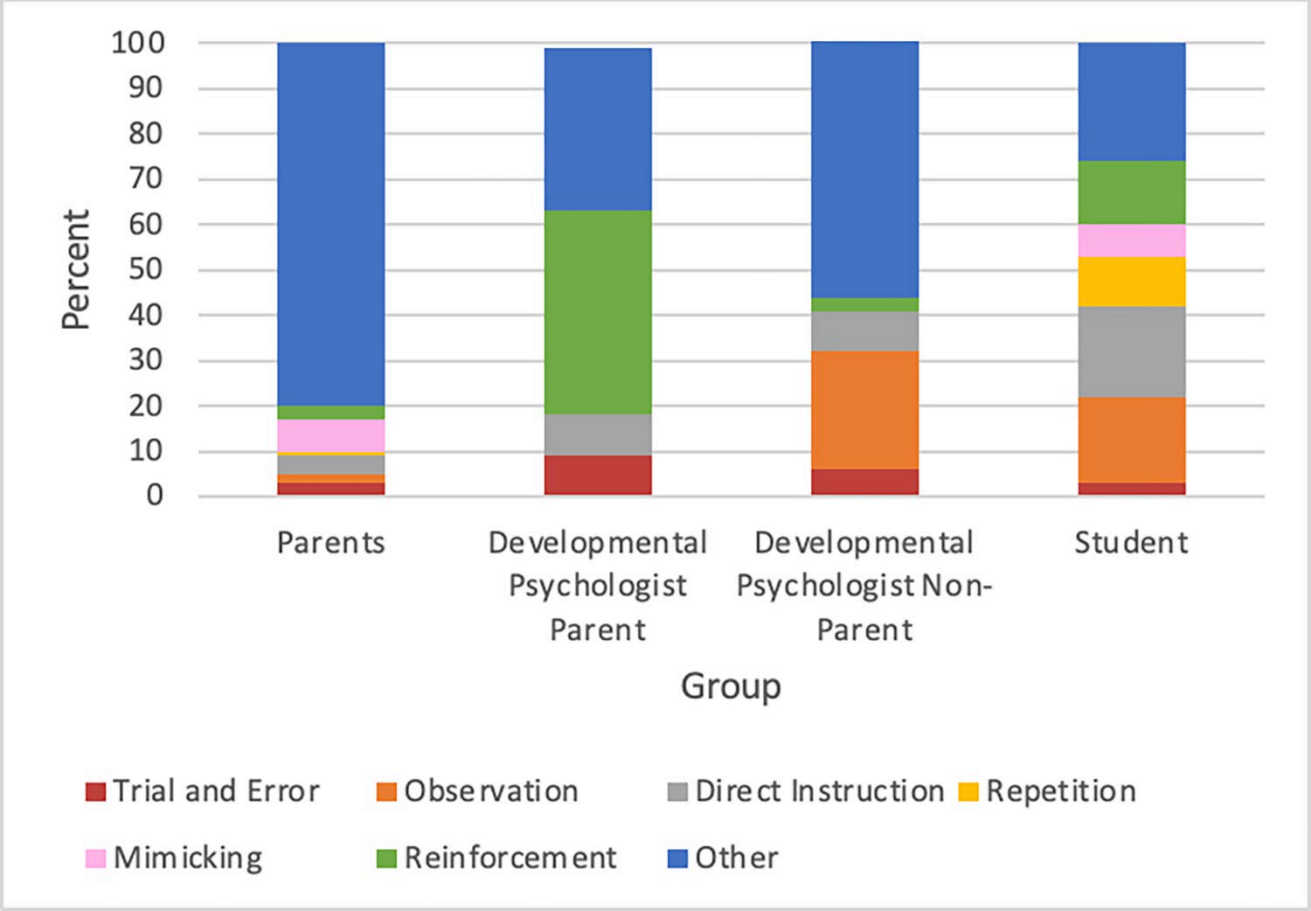
Participant responses on the mechanisms of development by group. *Note*: Statements not related to a mechanism were coded as Other.

The Mechanism category was created to distinguish between various learning mechanisms employed by children. Utterances that referred to mechanisms were relatively few (see Fig 5 and Table 3). In other words, participants did not mention many specific mechanisms that children use to learn. Therefore, most of this category was coded as “Other” across the groups. However, two noteworthy trends appeared: The Developmental Psychologist Parent group spent more time talking about reinforcement learning, while participants in the Developmental Psychologist Non-Parent and Student groups emphasized observational learning. In the Developmental Psychologist Parent group, 45 percent of their statements pertained to reinforcement (see Table 3). One participant in this group questioned her own involvement in her child’s word learning abilities through reinforcement:

“Yeah, I think she followed that, but I also wonder if we reinforced things like the shift from babbling to word learning in a way, like if she goes ’mamasa’, oh are you saying mama? And that’s like giving her cues from the environment to reinforce that coupling.”

In the Student groups, 19 percent of the utterances focused on observation as a source for child development and learning (see Table 3). In the Developmental Psychologist Non-Parent group, 26 percent of the utterances pertained to observation. For example:

“I think also observing what works for other people or what parents are demonstrating.”

## Discussion

Previous research on beliefs about how children develop has primarily assessed the attitudes and cognitions of parents via questionnaires with fixed questions and a fixed set of answers. While these studies have provided some evidence that parent’s beliefs affect how they interact with their children (e.g., [7, 8, 18]), the focus on parents and the use of questionnaires has limited our ability to understand the full scope of how people think and talk about child development. The current study used a series of focus groups to explore the nuances in how parents, non-parents, and specialists talk and think about child development. Overall, our findings suggest that 1) people are not always certain when making statements about how children develop, 2) parents, even those that are developmental psychologists, often use anecdotes to talk about child development, 3) people without children tend to think development is more controllable, and 4) people more often provide environment-based than genetic-based explanations for development.

### Certainty in beliefs about development

Certainty can be an index of the strength of beliefs (see, e.g., [21–23]), and thus it is notable that about 30% of statements reflected uncertainty for most groups. People with the most experience with children, the Developmental Psychologist Parents, exhibited the most certainty, with only 18% of statements coded as uncertain. This pattern of results aligns with research demonstrating that people who consider themselves more knowledgeable about an issue are more likely to hold stronger beliefs that are less malleable [24]. Additionally, the fact that the non-parent, non-expert group (Students) was the most uncertain in their statements has implications for educational interventions around child development. Beliefs that are held more confidently tend to be affectively stronger [25], and people who have stronger beliefs are more likely to hold on to those beliefs for a long time and be resistant to change [26]. Given that people are most uncertain in their beliefs about child development before they have experience with children, educational interventions may be more effective early in adulthood before ideas are entrenched.

The fact that participants were uncertain in many of their statements about child development also impacts how we interpret past work, particularly the statistical reliability of belief measurements in this domain. Participants who mark that they “agree” with a statement on a survey may be indicating a stable belief, or they may be making their best, uncertain guess at that moment. If researchers want to understand whether and how beliefs affect the ways in which adults interact with children in their daily lives, this distinction is important. We recommend that future studies measure certainty of belief for all questions.

### Statement type in adult discussions about development

We also found that parents used more anecdotes to support their reasoning, while developmental psychologists without children used the highest proportion of generic statements. It is possible that these developmental psychologists had less direct interaction with children than other groups and therefore had fewer anecdotes to pull from. Additionally, psychologists may be used to talking about child development more abstractly. Regardless of the reason, the difference in the use of anecdotes has implications for how beliefs may change with experience. As people interact with children more, their beliefs may become tied to their specific experiences rather than general information from books or conversations with friends. Anecdotes can be powerful methods of communication and persuasion, especially when culturally relevant. Researchers demonstrated that vivid anecdotes in the context of an experiment can shape beliefs and behaviors and may be particularly effective in communicating with vulnerable minority populations [27]. While it is possible that parents were merely using anecdotes to communicate their beliefs more clearly, the fact that parents used more anecdotes suggests that their experiences might drive their beliefs. This finding suggests that educational interventions with parents may be more effective if they are connected to lived experiences.

### Perceived control over child development

The third prominent finding is that non-parent groups (Student and Developmental Psychologist Non-Parent) discussed child development in more controllable terms than the parent groups (Parent and Developmental Psychologist Parent). Indeed, the percent of statements that expressed uncontrollability jumps from an average of 14% in non-parent groups to an average of 58.5% for parent groups. These findings may be the result of changing beliefs pre to postpartum. After having the experience of raising a child, mothers may be more skeptical of how much they actually can control regarding their child’s behavior and development. Mothers in the current study’s focus groups identified how their beliefs changed over time and addressed the challenges of raising a child, indicating a sense of uncontrollability:

“I was the same way, like I would be at Target and there would be a kid having a tantrum and I was like ‘that mom needs to get that kid under control’. Or like you’re on the airplane and there’s a crying baby and you’re like ‘oh my gosh the crying baby’. And now I’m like ‘oh my gosh that poor person’ because I’m like I’ve been there, it’s awful, there’s nothing you can do about it, you feel helpless, it’s a terrible feeling and I guess it’s just karma that, you know I’ve had to deal with the things that I was so judgmental about before I was living it.”

One previous study that focused on parent-infant interactions in relation to perceived control found that during free-play sessions at home, mothers who rated their infants as more difficult exhibited a directive parenting style and perceived a lower sense of control [28], providing an example of how adult beliefs about controllability affect parenting styles. If a mother believes that she can control her child’s future, she may try to oversee every aspect of her child’s life. Alternatively, if she decides that no matter what she does, she has no control over her child’s outcomes, she may not interfere as much, leaving her child to develop more independently. The stark difference we found in perceived control between parent and non-parent groups should be further explored, particularly considering the findings regarding the etiology of development.

### Beliefs about the etiology of child development

In terms of Etiology, the non-parent groups expressed more statements that indicated environmental causes of behavior. Similar to the theme of Statement Type, this finding may be indicative of the types of responses that more readily come to mind for each participant based on their experiences. Believing in genetic causes of behavior is connected to perceived uncontrollability [16], and thus the Parent and Developmental Psychologist Parent groups may have given more statements that indicated genetic causes to development because of their first-hand experience with how uncontrollable development can feel. Parents also may recall more instances where they see their child behaving similarly to themselves, which they may interpret as indicators of genetic influences. The potential connections between feelings of lack of control, perceived parent-child similarities, and beliefs in genetic origins of development should be explored further.

However, it is notable that consistent with the literature, several participants agreed that the environment and genetics often work in tandem [29]. The code level Interaction was developed for this purpose and may also be viewed as a way of being uncertain in one’s response:

“Yeah I think it’s complex, I mean I think there’s a part of me that always wants to think ‘oh yeah it’s mostly the environment’ but then sometimes you know I encounter certain things about cognitive development where I’m like ‘well there is that case scenario where you have two kids who maybe are totally different in their experiences and are doing similar things’ and that sometimes kind of pushes me a little bit to think about ‘well maybe there’s something there about genetics’ but you know, I don’t know.”

Combined with the results on controllability, this study sheds new light on Wang and Feigenson’s finding that people tend to give empiricist (or environment-based) accounts for the causes of development [15]. Wang and Feigenson concluded that humans have a bias toward an empiricists account of development. While we replicated, with a different methodology, the high proportion of empiricist statements, we also found high levels of uncertainty and a notable proportion of statements that expressed that genetic and environmental influences work in tandem. Thus, we found evidence that people may not staunchly endorse environmental influences as the main cause of development when given room to work through their thoughts. More generally, this finding supports our initial hypothesis that fixed-answer surveys may not be able to properly characterize beliefs about child development.

### Limitations

This study is an important addition to the current available research on adult beliefs about child development. However, the sample consisted of predominantly upper-middle class, white, educated Americans, thus the beliefs and themes that emerged from this study cannot be generalized beyond this population. Future studies should investigate how culture affects the ways that people talk about their beliefs about child development. An additional limitation derives from the nature of focus groups. While these types of semi-structured group interviews help to gather a large amount of information efficiently, they pose a chance for certain individuals to dominate the discussion, thus creating an environment that makes it difficult for others to voice their differing opinions [30]. Furthermore, the focus groups in this study ranged from 2 to 13 participants per group, so future studies should use more homogeneous group sizes. Our study used a small sample of focus groups with the intention of designing a survey that reflects the language and topics that people use in the real world. Future studies should collect more data from these groups with larger sample sizes. Additionally, the moderator used strategies to encourage participation from all group members, however, each session varied depending on the participants’ involvement. Follow-up studies will use other methods to corroborate the current findings.

## Conclusion

Focus groups are beneficial as a method of qualitative data collection because they allow researchers to efficiently gather a large amount of information on a topic and can help to generate novel hypotheses. Additionally, focus groups may assist in designing survey materials that reflect the language and topics that people use in the real world. In this study, we identified five major themes regarding the way that people talk about cognitive development: Certainty, Statement Type, Control, Etiology, and Mechanism. These themes provide insight into where adult beliefs about child development stem from. For example, we found that parents are more likely to use anecdotes to describe child development and are also more likely to believe that development is not entirely controllable and has a genetic component. This pattern of results suggests that personal experience with children changes not only beliefs, but the evidence used to form a belief. Combined with our finding that many statements on development expressed uncertainty, our data suggest that attitudes about child development may be highly malleable, particularly at the transition to parenthood when beliefs may be re-forming.

While this work may complicate our understanding of previous literature, it leads to three concrete suggestions for future work and survey development. First, researchers should measure certainty when assessing beliefs about child development: if parents only weakly agree with a given statement about development, they may not act on their belief. Second, researchers should explore whether and when beliefs about child development are malleable. We found some evidence that beliefs about child development change in quality across life experiences and given the evidence that beliefs about development affect how people interact with children, it is important to know precisely how and when these beliefs change. Lastly, our findings highlight the differences in beliefs about child development across different levels of expertise and experience with children. Most research on beliefs about child development is conducted on parents [31], and yet the development of children in society is affected not only by parent attitudes, but also by the community’s beliefs and priorities. From voting on policy decisions and institutional priorities to everyday interactions with children and parents, all members of society influence how children are raised. Therefore, we as scientists should expand our focus to study beliefs about child development not just in parents, but in society at large.

## Supporting information

S1 File Focus group questions. (PDF)
