# Supplementary material for: Adult beliefs about cognitive development vary across experience and expertise: A focus group study
Source: PLoS One. 2022 Aug 1;17(8):e0272254. doi: 10.1371/journal.pone.0272254 (PMC9342719; doi:10.1371/journal.pone.0272254)
Supplement: S1 File — (PDF) [file pone.0272254.s001.pdf]

S1 File. Focus Group Questions

1. Describe a recent experience you had with children
2. How do you believe young children develop early cognitive skills, such as reasoning and problem solving?
    a. Do you believe that cognitive development follows a certain path or trajectory? In other words, do children move progressively from one stage to the next, or do they sometimes go backwards or skip steps?
    b. How do you think that genetics influence cognitive development?
    c. How do you think that the environment influences cognitive development?
3. How do you believe young children learn language? (for example: sounds, what words mean, grammar, etc.)
    a. Do you believe that language development follows a certain path or trajectory? In other words, do children move progressively from one stage to the next, or do they sometimes go backwards or skip steps?
    b. How do you think that genetics influence language development?
    c. How do you think that the environment influences language development?
4. How do you believe young children learn their first words? For example, how do children figure out what dog means?
    a. How do children learn more abstract words like “more” or “idea”?
    b. How do you think that genetics influence word learning?
    c. How do you think that the environment influences word learning?
5. How has your experience with children affected the way in which you think that they develop? *(Students only)*
6. How has your experience with your own children affected the way in which you think that they develop? *(Parents only)*

7. How has your experience researching child development affected the way in which you think that they develop? *(DP_Ps and DP_NPs only)*
    a. Is there an experience that led to that belief?
8. What do you consider the most important elements of this discussion?
